# Preclinical Evaluation of Oncolytic Vaccinia Virus for Therapy of Canine Soft Tissue Sarcoma

**DOI:** 10.1371/journal.pone.0037239

**Published:** 2012-05-15

**Authors:** Ivaylo Gentschev, Marion Adelfinger, Rafael Josupeit, Stephan Rudolph, Klaas Ehrig, Ulrike Donat, Stephanie Weibel, Nanhai G. Chen, Yong A. Yu, Qian Zhang, Martin Heisig, Douglas Thamm, Jochen Stritzker, Amy MacNeill, Aladar A. Szalay

**Affiliations:** 1 Genelux Corporation, San Diego Science Center, San Diego, California, United States of America; 2 Department of Biochemistry, University of Wuerzburg, Wuerzburg, Germany; 3 Rudolf Virchow Center for Experimental Biomedicine, University of Wuerzburg, Wuerzburg, Germany; 4 Institute for Molecular Infection Biology, University of Wuerzburg, Wuerzburg, Germany; 5 Department of Radiation Oncology, Moores Cancer Center, University of California San Diego, La Jolla, California, United States of America; 6 Institut für Medizinische Strahlenkunde und Zellforschung (MSZ), University of Wuerzburg, Wuerzburg, Germany; 7 Department of Clinical Sciences, College of Veterinary Medicine and Biomedical Sciences, Colorado State University, Fort Collins, Colorado, United States of America; 8 Department of Pathobiology, College of Veterinary Medicine, University of Illinois, Urbana, Illinois, United States of America; University of Chicago, United States of America

## Abstract

Virotherapy using oncolytic vaccinia virus (VACV) strains is one promising new strategy for canine cancer therapy. In this study we describe the establishment of an *in vivo* model of canine soft tissue sarcoma (CSTS) using the new isolated cell line STSA-1 and the analysis of the virus-mediated oncolytic and immunological effects of two different Lister VACV LIVP1.1.1 and GLV-1h68 strains against CSTS. Cell culture data demonstrated that both tested VACV strains efficiently infected and destroyed cells of the canine soft tissue sarcoma line STSA-1. In addition, in our new canine sarcoma tumor xenograft mouse model, systemic administration of LIVP1.1.1 or GLV-1h68 viruses led to significant inhibition of tumor growth compared to control mice. Furthermore, LIVP1.1.1 mediated therapy resulted in almost complete tumor regression and resulted in long-term survival of sarcoma-bearing mice. The replication of the tested VACV strains in tumor tissues led to strong oncolytic effects accompanied by an intense intratumoral infiltration of host immune cells, mainly neutrophils. These findings suggest that the direct viral oncolysis of tumor cells and the virus-dependent activation of tumor-associated host immune cells could be crucial parts of anti-tumor mechanism in STSA-1 xenografts. In summary, the data showed that both tested vaccinia virus strains and especially LIVP1.1.1 have great potential for effective treatment of CSTS.

## Introduction

Canine soft tissue sarcomas (CSTSs) typically arise in middle-age to old dogs and are a diverse group of cancers that collectively comprise 7% of cutaneous and 15% of subcutaneous canine cancers [1]–[5]. The annual incidence of CSTSs is about 35 per 100,000 dogs at risk [4]. Although CSTSs have similarity in histological features and clinical behavior, these tumors are phenotypically diverse with frequently controversial histogenesis [6]. They include fibrosarcomas, myxosarcomas, liposarcomas, perivascular wall tumors, peripheral nerve sheath tumors (PNST), pleomorphic sarcoma, mesenchymoma, leiomyosarcoma and rhabdomyosarcomas [1], [2], [6], [7]. CSTSs are graded as low (grade I), intermediate (grade II) and high (grade III) grade tumors based on mitotic index, tumor necrosis, and degree of differentiation [6], [8], [9]. Histological grading is considered a prognostic indicator for CSTSs [10]. Grade I soft tissue sarcomas tend to be locally invasive but rarely metastasize, whereas grade II tumors are invasive and have a 7–33% chance of spreading to the lung or regional lymph nodes [6]. Grade III tumors are uncommon and are thought to have a higher rate of recurrence and metastasis [6].

Treatment routines consist of wide surgical excision, radiation therapy and adjuvant chemotherapy. Despite progress in the diagnosis and treatment of CSTSs, the prognosis for canine patients with high-grade soft tissue sarcoma is poor due to the high probability of metastasis [1], [2], [11]. Therefore, the development of new therapies for CSTSs is very important. One of the most promising novel cancer therapies is oncolytic virotherapy. This method is based on the capacity of oncolytic viruses (OVs) to eliminate malignancies by direct targeting and lysis of cancer cells. Currently several OV platforms (herpes simplex virus, vaccinia virus, Seneca valley virus and reovirus) are in or entering Phase III human clinical trials. In addition, in China the oncolytic adenovirus H101 has been approved in the treatment of human patients with head and neck cancer since 2005 [12].

In this study, we analyzed the therapeutic potential of two different oncolytic vaccinia virus strains against CSTSs in a preclinical mouse model. Two tested viruses, namely GLV-1h68 and LIVP1.1.1 contain an inactive thymidine kinase (*tk*) gene and show inherently more tumor-selective replication than vaccinia virus WR strain [13], [14]. LIVP1.1.1 was isolated from a wild type stock of Lister strain of vaccinia virus (Lister strain, Institute of Viral Preparations, Moscow, Russia). We have chosen LIVP1.1.1 for this study, since it was less virulent compared to other Lister strain isolates (Chen et al manuscript in preparation).

The virus strain GLV-1h68 was engineered by inserting 3 expression cassettes encoding a) *Renilla* luciferase-green fluorescent protein (Ruc-GFP) fusion protein into the F14.5L locus, b) ß-galactosidase into the thymidine kinase (tk) locus, and c) ß-glucuronidase into the hemagglutinin locus from the genome of the LIVP strain [13]. GLV-1h68 showed potent anticancer efficacy in many different human tumor xenograft models, including human breast cancer [13], anaplastic thyroid carcinoma [15], [16], malignant pleural mesothelioma [17], pancreatic tumor [18], hepatocellular carcinoma (HCC) [19], prostate carcinoma [20], and squamous cell carcinoma [21]. Moreover, results of a Phase 1 study of intravenous administration of GL-ONC1 (GLV-1h68) vaccinia virus in human patients with advanced solid cancer demonstrated safety, preliminary evidence of anticancer activity and virus replication (http://www.ncri.org.uk/ncriconference/2010abstracts/abstracts/C122.htm).

In addition, we have already demonstrated the therapeutic effect of GLV-1h68 against canine mammary adenoma and carcinoma using ZMTH3 and MTH52c cells, respectively, in xenograft models [22], [23].

Here, we describe the establishment of an *in vivo* model of CSTS using a newly isolated cell line derived from a canine patient with a low grade II soft tissue sarcoma. The development of this new xenograft model was necessary, because very few canine soft tissue sarcoma cell lines exist [24]. In addition, we analyzed the virus-mediated oncolytic and immunological effects of two different Lister VACV strains against CSTS by using fluorescence imaging, immunohistochemistry and flow cytometry.

## Materials and Methods

### Ethics statement

All animal experiments were carried out in accordance with protocols approved by the Institutional Animal Care and Use Committee of Explora Biolabs (San Diego, CA) and/or the government of Unterfranken, Germany (permit number: 55.2-2531.01-17/08).

### Donor

The cell line STSA-1 was derived from a tumor of a seven-year-old, male, neutered golden retriever dog that presented with a firm, painful, erythematous mass on the left forelimb. The mass was surgically debulked with excision of the deep digital flexor and flexor carpi muscles as they were extensively infiltrated by the tumor. Histopathology performed on the surgically excised mass was described in Results. The patient underwent full course radiation therapy. At a three month recheck examination, thoracic radiographs and abdominal ultrasound showed no evidence of metastasis, but radiographs of the left front limb revealed severe lysis of the distal ulna, caudal distal radius, and carpal bones. A fine needle aspirate of an enlarged left prescapular lymph node was diagnosed by cytology as metastatic meschenchymal neoplasia. At this time, the limb was amputated and enlarged lymph nodes were removed. Cells were isolated aseptically from the mass for culture in the laboratory as described in Materials and Methods. Cytochemical staining and genetic analysis of the isolated canine cells were performed as described below.

The entire limb as well as axillary and prescapular lymph nodes were formalin-fixed and submitted for histology. Histologically, the lesion was consistent with a soft tissue sarcoma of intermediate grade with vascular invasion, infiltration of the tumor cells into the bone marrow cavity and metastases to draining lymph nodes. Over the next two months, the canine patient received chemotherapy, but regrowth of the mass was observed and the dog was euthanized due to side effects caused by the chemotherapeutics.

### Cell culture

African green monkey kidney fibroblasts (CV-1) were obtained from the American Type Culture Collection (ATCC). CV-1 cells were cultured in DMEM supplemented with antibiotic-solution (100 U/ml penicillin G, 100 units/ml streptomycin) and 10% fetal bovine serum (FBS; Invitrogen GmbH, Karlsruhe, Germany).

STSA-1 cells described in this paper were isolated from surgically excised spontaneous canine tumor removed from a patient at the University of Illinois Veterinary Teaching Hospital. Fat and necrotic tissue were removed from an unfixed section of the tumor. The mass was then minced into one millimeter cubes, placed in a 25 cm^2^ cell culture flask (Nunc, Wiesbaden, Germany) and left to adhere for 10 min at room temperature with subsequent addition of minimum essential medium with Earle's salts supplemented with 2 mM glutamine, 50 U/mL penicillin G, 50 µg/mL streptomycin, 1 mM sodium pyruvate, 0.1 mM nonessential amino acids (MEM-C), and 10% FBS, then incubated at 37°C, 5% CO_2_, and 100% humidity. Trypsinization and passage of the cultured cells was performed when cells covered approximately 80% of the flask surface or sooner if tissue explants were decaying. Once a primary culture was established, it was maintained in MEM-C with 10% FBS and incubated as above.

To analyze cell type using cytochemical and immunocytochemical stains, primary cells were grown in 35 mm diameter plates (Nunc) as described above. Then cells were trypsinized, collected in MEM-C with 10% FBS and pelleted by centrifugation at 400×g for 5 min. The cellular pellets were suspended in 1 mL phosphate-buffered saline (PBS) from which 100 µL aliquots of cells were cytocentrifuged at 1000 rpm for 3 min onto charged glass slides. Subsequently, the adherent cells were incubated for 10 minutes with BCIP/NBT phosphatase substrate (KPL, Inc., Gaithersburg, Maryland, USA) to detect alkaline phosphatase (ALP) activity or were immunostained directly with a murine anti-canine CD18 monoclonal antibody (CA16.3C10, a gift from Dr. Peter Moore, University of Califorina, Davis, CA, USA). Additional slides were subjected to antigen retrieval in a decloaking chamber (Biocare Medical, Concord, CA, USA) and incubated with a cocktail of murine monoclonal anti-AE1 and anti-AE3 antibodies (Biogenex, San Ramon, CA, USA) to detect cytokeratin, or with a murine monoclonal anti-vimentin antibody (V-9, Biogenex).

Following successful cell line establishment, cells were confirmed to be of canine origin and identity validated through multiplex species-specific PCR and short tandem repeat analysis, as recently described [25].

### Virus strains

GLV-1h68 is a genetically stable oncolytic virus strain designed to locate, enter, colonize and destroy cancer cells without harming healthy tissues or organs [13].

LIVP1.1.1 was derived from LIVP (Lister strain, Institute of Viral Preparations, Moscow, Russia). The sequence analysis of LIVP1.1.1 revealed the presence of different mutations in several genes including that of thymidine kinase (Chen et al manuscript in preparation). In addition, LIVP1.1.1 demonstrated also different plaque form morphology in comparison to GLV-1h68 in CV-1 cells.

### Cell viability assay

8×10^4^ cells/well were seeded in 24-well plates (Nunc, Wiesbaden, Germany). After 24 h in culture, cells were infected with either LIVP1.1.1 or GLV-1h68 using multiplicities of infection (MOI) of 0.1 and 1.0. The cells were incubated at 37°C for 1 h, then the infection medium was removed and subsequently the cells were incubated in fresh growth medium. The amount of viable cells after infection was measured using 3-(4,5-dimethylthiazol-2-yl)-2,5-diphenyltetrazolium bromide (MTT) (Sigma, Taufkirchen, Germany). At 24, 48, 72, or 96 h after infection of cells, medium was replaced by 0.5 ml MTT solution at a concentration of 2.5 mg/ml MTT dissolved in RPMI 1640 without phenol red and incubated for 2 h at 37°C in a 5% CO_2_ atmosphere. After removal of the MTT solution, the color reaction was stopped by adding 1 N HCl diluted in isopropanol. The optical density was then measured at a wavelength of 570 nm. Uninfected cells were used as reference and were considered as 100% viable.

### Viral replication

For the viral replication assay, cells grown in 24-well plates were infected with either LIVP1.1.1 or GLV-1h68 at an MOI of 0.1. After one hour of incubation at 37°C with gentle agitation every 20 min, the infection medium was removed and replaced by a fresh growth medium. After 1, 6, 24, 48, 72 and 96 hours, the cells and supernatants were harvested. Following three freeze-thaw cycles, serial dilutions of the lysates were titered by standard plaque assays on CV-1 cells. All samples were measured in triplicate.

### Vaccinia virus-mediated therapy of STSA-1 xenografts

Tumors were generated by implanting 1×10^6^ canine soft tissue sarcoma cells in 100 µl PBS subcutaneously into the right hind leg of 6- to 8-week-old female nude mice (NCI/Hsd/Athymic Nude-*Foxn1* ^nu^, Harlan Winkelmann GmbH, Borchen, Germany). On day 28, a single dose of LIVP1.1.1 or GLV-1h68 virus (1×10^7^ plaque forming units [pfu] in 100 µl PBS) was injected into the tail vein intravenously (i.v.). The control animals were injected i.v. with PBS only. Tumor growth was monitored weekly in two dimensions using a digital caliper. Tumor volume was calculated as [(length×width^2^)/2].

The significance of the results was calculated by two-way analysis of variance (ANOVA) or Kaplan-Meier and log-rank (Mantel-Cox) tests (GraphPad Prism software, San Diego, USA) or Student's t-test. Results are displayed as means ± s.d. (standard deviation). P values of <0.05 were considered significant.

### Histological analysis of tumors

The spontaneous canine tumor surgically removed from a patient at the University of Illinois Veterinary Teaching Hospital was submitted to the University of Illinois Veterinary Diagnostic Laboratory (VDL) for histopathologic analysis. Portions of the tissues were paraffin-embedded, sectioned and stained with hematoxylin and eosin (H&E) to identify the tumor type.

For histological studies of xenograft model, tumors were excised and snap-frozen in liquid N_2_, followed by fixation in 4% paraformaldehyde/PBS at pH 7.4 for 16 h at 4°C. Tissue samples were sectioned (10 µm thickness) with the cryostat 2800 Frigocut (Leica Microsystems GmbH, Wetzlar, Germany). After dehydration in 10% and 30% sucrose (Carl Roth, Karlsruhe, Germany) specimens were embedded in Tissue-Tek® O.C.T. (Sakura Finetek Europe B.V., Alphen aan den Rijn, Netherlands).

Endothelial blood vessel cells were stained with a hamster monoclonal anti-CD31 antibody (Chemicon International, Temecula, USA; MAB1398Z). Anti-Mouse Ly-6G (eBioscience, San Diego, USA; 14-5931-81) was used to stain neutrophil granulocytes. DyLight549- and DyLight649-conjugated secondary antibodies (donkey) were obtained from Jackson ImmunoResearch (Pennsylvania, USA).

A part of tissue sectioning was performed as described by [26]. In this case, VACVs were labeled using polyclonal rabbit anti-vaccinia virus (anti-VACV) antibody (Abcam, Cambridge, UK), which was stained using Cy3-conjugated donkey anti-rabbit secondary antibodies obtained from Jackson ImmunoResearch (West Grove, PA, USA).

Immune cells were labeled using rat anti-mouse MHCII antibody detecting a polymorphic determinant present on B cells, monocytes, macrophages and dendritic cells (eBioscience, San Diego, CA) and Cy5-conjugated secondary antibodies (donkey) obtained from Jackson ImmunoResearch (West Grove, PA, USA).

The fluorescence-labeled preparations were examined using the MZ16 FA Stereo-Fluorescence microscope (Leica) equipped with the digital DC500 CCD camera and the Leica IM1000 4.0 software (1300×1030 pixel RGB-color images). Digital images were processed with Photoshop 7.0 (Adobe Systems, Mountain View, CA, USA).

### Measurement of blood vessel density and fluorescence intensity of the CD31 signal in the tumor tissue

Blood vessel density was measured in digital images (×100 magnification) of CD31-labelled 10-µm-thick tumor cross-sections using Leica IM1000 4.0 software. Eighteen images per tumor were analyzed per staining (3 tumors per group, 3 sections of each tumor and 6 images per section). Exposure time for individual images was adjusted to ensure clear visibility of all detectable blood vessels and decorated with 6 equidistant horizontal lines using Photoshop 7.0. All blood vessels crossing these lines were counted to obtain the vessel density per section.

Fluorescence intensity of the CD31-labelling in 10-µm-thick sections of control tumors and infected areas of virus-colonized tumors was measured on digital images (×100 magnification) of specimens stained for CD31 immunoreactivity. On the fluorescence microscope, the background fluorescence was set to a barely detectable level by adjusting the gain of the CCD camera before all the images were captured with identical settings. RGB-images were converted into 8-bit gray scale images (intensity range 0–255) using Photoshop 7.0. The fluorescence intensity of the CD31-labelling represented the average brightness of all vessel-related pixels and was measured using Image J software http://rsbweb.nih.gov/ij.

### Flow cytometric (FACS) analysis

For flow cytometric analysis, three or four mice from each group were sacrificed by CO_2_ inhalation and the tumors were removed. The tumor tissues were minced and incubated individually in 10.000 CDU/ml Collagenase I (Sigma, Steinheim, Germany) and 5 MU/ml DNase I (Calbiochem, Darmstadt, Germany) for 75 min at 37°C and then passed through a 70-µm nylon mesh filter (BD Biosciences, Erembodegem, Belgium).

To block non-specific staining, single cells were preincubated with 0.5 µg of anti-mouse CD16/32 antibody (clone 93, Biolegend, San Diego, USA) per one million cells for 20 min on ice. After that, the cells were incubated at 4°C for 10 min in PBS with 2% FCS in the presence of appropriate dilutions of labeled monoclonal antibodies: anti-mouse MHCII-PE (Clone M5, eBioscience, San Diego, CA, USA), anti-CD11b-PerCPCy5.5 (Clone M1/70, eBioscience), anti-CD11c-APC (Clone N418, BioLegend, San Diego, USA), anti-CD49b-APC (Clone DX5, BioLegend), anti-CD19-PerCP-Cy5.5 (Clone 6D5, BioLegend), anti-F4/80-APC (Clone BM8, eBioscience), anti-Ly-6G-PE (Clone 1A8, BD Biosciences) and anti-CD45-PerCP (Clone 30-F11, BD Biosciences).

Numbers of neutrophils in tumors and peripheral blood of STSA1-tumor-bearing mice were determined in parallel by staining with APC-eFluor 780-conjugated mAbs to Ly-6G (Clone RB6-8C5 eBioscience, San Diego, CA, USA). Before specific Ab staining, cells were incubated with Fc blocker (anti-CD16/CD32 mAb) for 20 min.

Stained cells were subsequently analyzed, using an Accuri C6 Cytometer and FACS analysis software CFlow Version 1.0.227.4 (Accuri Cytometers, Inc. Ann Arbor, MI USA).

## Results

### Histological and pathological examination of original canine tumor and xenograft tumors

**Figure 1:**
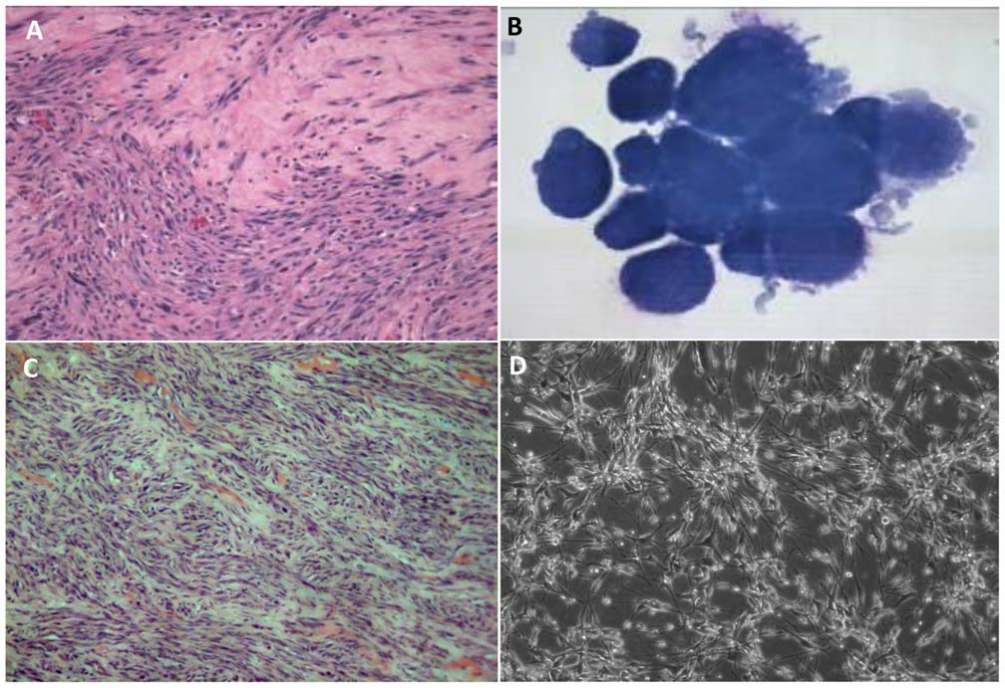
Imaging of STSA-1 tumors (A, C) and canine soft tissue sarcoma cells (B, D). (**A**) Histologic section of a canine soft tissue sarcoma from the limb of a golden retriever dog, hematoxylin and eosin stain (H&E, ×200 magnification). (**B**) Cytology of canine soft tissue sarcoma cells STSA-1 isolated from a subcutaneous mass on a golden retriever dog, Wright-Giemsa stain (×1000 magnification). (**C**) Canine soft tissue sarcoma STSA-1 xenograft, right flank, athymic nude mouse (H&E, ×200 magnification). (**D**) Transmitted light microscopy of uninfected STSA-1 cells in MEM-C culture (×100 magnification).

The histological examination of the original primary tumor revealed atypical mesenchymal cells haphazardly arranged in bundles and streams (Fig. 1A). The cells had a moderate amount of eosinophilic cytoplasm and oval nuclei with coarse, clumped chromatin and one to two prominent nucleoli. Moderate anisocytosis and anisokaryosis were noted. There were one to two mitotic figures per ×400 magnification. Neoplastic cells extended into adjacent muscle and areas of compacted collagen. The mass was diagnosed as a low grade II soft tissue sarcoma with narrow surgical margins. In addition, aliquots of canine cell explants from the tumor (in following termed as STSA-1) were analysed by cytochemical staining as described above (Fig. 1B, Table 1 and Figure S1). Alkaline phosphatase (ALP) activity is detectable in cells derived from bone, liver, kidney and intestine; CD18 is a marker for histiocytic cells; cytokeratin is found within cells of epithelial origin; and vimentin is present in mesenchymal cells. Cells were positive for vimentin, but negative for other proteins, which supports the soft tissue sarcoma diagnosis (Table 1 and Figure S1). The canine origin of STSA-1 cells was also confirmed through multiplex species-specific microsatellite PCR (Figure S2).

**Table 1:** Staining characteristics of cells isolated from a spontaneous tumor growth on a golden retriever dog.

Cell Name	Histopathological Diagnosis	Positive Cytochemical Staining	Negative Cytochemical Staining
STSA-1	Soft tissue sarcoma	Vimentin	Alkaline phosphatase CD18 Cytokeratin

To analyze the tumorigenic potential of the new isolated cell line STSA-1, 1×10^6^ cells were implanted into the right hind leg of 6- to 8-week-old female nude mice. Ninety-six percent (58 from 60) of the STSA-1-implanted mice developed a detectable tumor mass. Seven to nine weeks post implantation, most mice developed tumors with volumes of 2500 to 3000 mm^3^. None of these mice showed any signs of metastasis or of invasive growth pathologically. Histological analysis of the tumors revealed a population of cells with numerous signs of malignancy including mitotic figures, anisokaryosis, anisocytosis, binucleation and multinucleation (Fig. 1C). In cell culture STSA-1 cells are spindle-shaped with long extensions and do not form closed monolayers (Fig. 1D).

The data demonstrated that the STSA-1 cell line is highly tumourigenic in female nude mice and also mimicked the behavior of a soft tissue sarcoma.

### Analysis of the oncolytic potential of VACV strains against canine sarcoma cells in culture

**Figure 2:**
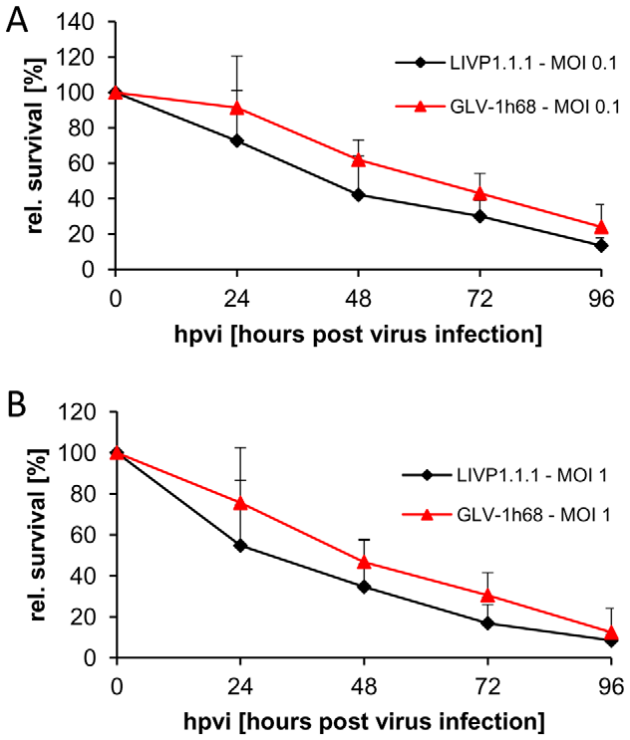
Viability of canine soft tissue sarcoma cells after LIVP1.1.1 or GLV-1h68 infection at MOIs of 0.1 (A) and 1.0 (B), respectively. Viable cells after infection were detected using 3-(4, 5-dimethylthiazol-2-yl)-2,5-diphenyltetrazolium bromide (MTT) (Sigma, Taufkirchen, Germany). Mean values (n = 3) and standard deviations are shown as percentages of respective controls. The data represent two independent experiments. There were no significant differences between groups (P>0.05).

STSA-1 cells were seeded three days prior to infection in 24-well plates. Cells were then infected with either LIVP1.1.1 or GLV-1h68 at MOIs of 1.0 and 0.1, respectively. Cell viability was analyzed at 24, 48, 72 and 96 hours post-virus-infection (hpvi) by MTT-assays (Fig. 2). The data demonstrated that both VACV strains efficiently infected and destroyed cells of the canine soft tissue sarcoma line STSA-1 under cell culture conditions.

Although there was no statistically significant difference in number of viable cells between the two virus strains, these results indicated that LIVP1.1.1 virus infection led to a somewhat more efficient eradication of the canine sarcoma cells in culture compared to GLV-1h68- treatment.

### Efficacy of LIVP1.1.1- and GLV-1h68-replication in STSA-1 cells

**Figure 3:**
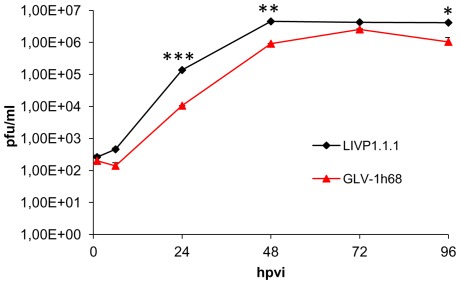
Comparison of the replication capacity of the vaccinia virus strains GLV-1h68 and LIVP1.1.1 in canine soft tissue sarcoma cells. For the viral replication assay, STSA-1 cells grown in 24-well plates were infected with either GLV-1h68 or LIVP1.1.1 at an MOI of 0.1. Cells and supernatants were collected for the determination of virus titer at various time points. Viral titers were determined as pfu per well in triplicates by standard plaque assay in CV-1 cell monolayers. Averages plus standard deviation are plotted. The data represent two independent experiments. The statistical significance was analyzed using two-way ANOVA followed Bonferroni post-test on log transformed PFU data. *, **, and *** indicate P<0.05, 0.01, and 0.001, respectively.

STSA-1 cells were infected with either LIVP1.1.1 or GLV-1h68 at a MOI of 0.1. Standard plaque assay was performed for all samples to determine the viral titers at different time points during the course of infection (Fig. 3). The maximum viral titers (total) of each virus were determined for LIVP1.1.1 (4.54×10^6^ pfu/well) at 48 hpvi and for GLV-1h68 (2.55×10^6^ pfu/well) at 72 hpvi. In addition, we found significant differences from titers of LIVP1.1.1 compared with GLV-1h68 at 24 hpvi (***P<0.001) and 48 hpvi (**P<0.01) as well as at 96 hpvi (*P<0.05).

These data demonstrated that LIVP1.1.1 can replicate more efficiently than GLV-1h68 in STSA-1 cells under these experimental conditions.

### Oncolytic effect of a single systemic application of VACVs on STSA-1 xenografts

**Figure 4:**
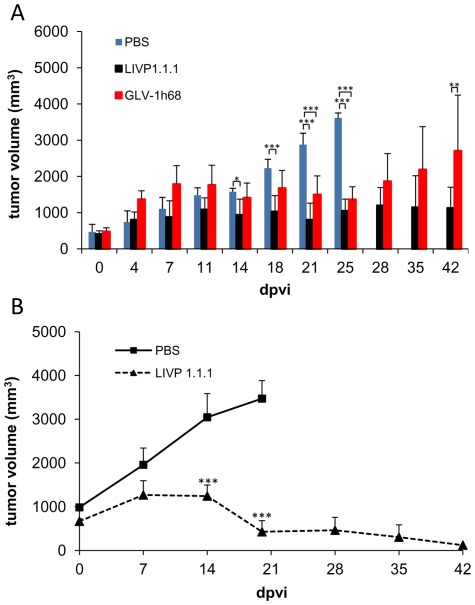
Growth of canine soft tissue sarcoma tumors in virus-and mock-treated mice. (**A**) Groups of STSA-1 tumor-bearing nude mice (n = 6) were either treated with a single dose of 1×10^7^ pfu GLV-1h68, LIVP1.1.1 or with PBS (mock control). Tumor size was measured twice a week. Two-way analysis of variance (ANOVA) with Bonferroni post-test was used for comparison of two corresponding data points between groups. *, **, and *** indicate P<0.05, 0.01, and 0.001, respectively. (**B**) Animals with established STSA-1 flank tumors (>600 mm^3^) were distributed into two experimental groups (n = 4 per group). Flank tumors were treated with injections of a single dose of LIVP1.1.1 (1×10^7^ pfu) or PBS alone as control. The statistical significance was confirmed by Student's t-test (***p<0.001).

Eighteen female nude mice at an age of 6–8 weeks were implanted with 1×10^6^ STSA-1 cells. Four weeks post implantation, all mice developed tumors with volumes of 400 to 500 mm^3^. Animals were separated into three groups (n = 6) and were injected with a single dose of GLV-1h68, LIPV1.1.1 (1×10^7^ pfu in 100 µl PBS) or PBS (100 µl) intravenously (i.v.) into the lateral tail vein. Tumor size was measured twice a week. As shown in Fig. 4A, the virus treatment led to a significant difference (***P<0.001) in tumor growth between PBS controls and all virus-treated mice on 21 and 25 days post-virus-injection (dpvi). Due to excessive tumor burden (>3000 mm^3^), all animals of the control group were euthanized after 25 dpvi. In addition, at 42 dpvi we determined a significant difference (**P<0.01) between GLV-1h68 vs LIVP1.1.1. Moreover, two of six mice of the GLV-1h68-treated group developed tumors with volumes greater than 3000 mm^3^ and had to be euthanized after 42 dpvi.

These data indicated that LIVP1.1.1 had a higher oncolytic potential than GLV-1h68 against canine soft tissue sarcoma xenografts.

To determine whether the initial tumor size at the time of virus delivery would affect the outcome of virotherapy, we started a second therapeutic experiment in which the average starting tumor volume before injection was 40% larger compared to that of the corresponding LIVP1.1.1-group in the experiment described before (see Fig. 4A). In this experimental setting, a single i.v. injection of LIVP1.1.1 into mice bearing canine soft tissue sarcoma STSA-1 xenografts led to near-complete tumor regression over a 42-day period without toxicity (Fig. 4B). These data could be evidence, that larger STSA-1-tumors (600 to 1000 mm^3^) are more responsive to LIVP1.1.1-treatment than smaller STSA-1-tumors under these experimental conditions.

**Figure 5:**
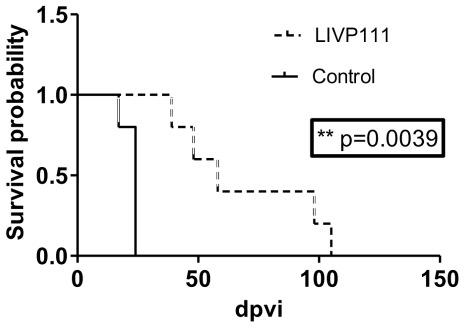
Survival curves of LIVP1.1.1-treated and non-treated STSA-1 tumor-bearing mice. The comparison of the survival between the different treatment groups (n = 5) was statistically evaluated by Kaplan-Meier and log-rank (Mantel-Cox) tests (GraphPad Prism, San Diego, CA). P<0.05 was considered statistically significant. ** P = 0.0039.

In a third independent therapeutic experiment we analyzed the long-term survival of LIVP1.1.1-treated mice compared to PBS-treated control mice (Fig. 5). Here, the mice of the untreated group were euthanized at day 17 (n = 1) and day 24 (n = 4), due to the development of tumors with volumes greater than 3000 mm^3^. In the virus-treated group, two animals were found dead at 39 and 58 dpvi, respectively (no pathological changes were observed). The remaining three animals of this group were euthanized under certain criteria, 30% weight loss (n = 2; at days 48 and 98) and tumor greater volume than 3000 mm^3^ (n = 1; at day 105).

In summary, this experiment demonstrated that a single injection with LIVP1.1.1 vaccinia virus led to significantly longer survival (**P = 0.0039) of the treated mice compared to the non-treated animals.

### Comparison of LIVP1.1.1 and GLV-1h68 distribution in tumor-bearing nude mice

**Figure 6:**
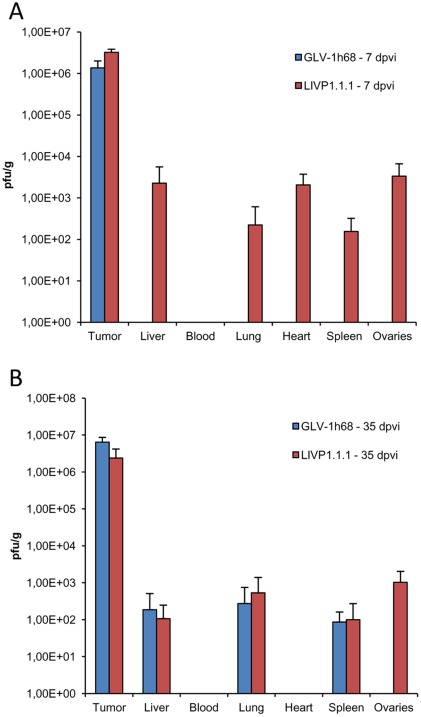
Virus distribution in STSA-1 xenografts after 7 (A) and 35 (B) day post injection with GLV -1h68 or LIVP1.1.1. Tumor-bearing mice were injected with 1×10^7^ pfu of GLV-1h68 or LIVP1.1.1. Three mice of each group were analyzed at 7 and 35 dpvi for virus distribution. The data were determined by standard plaque assays on CV-1 cells using aliquots of the homogenized tissue and were displayed as mean pfu/g organ or tissue (n = 3). For each organ, aliquots of 0.1 ml were measured in triplicates (detection limit: 10 pfu/organ or tissue). The values are the mean of triplicate samples, and the bars indicate SD. Statistical analysis for the tumors was performed using 1-way ANOVA followed by Bonferroni post-test on log transformed pfu data. There were no significant differences between groups (P>0.05). The study was repeated in two independent experiments.

In order to analyze the reason for different oncolytic effects of VACV strains in STSA-1 xenografts we first compared the virus colonization and distribution pattern of the virus strains *in vivo* at early and late time points after virus treatment. Figure 6 summarizes the virus distribution data in sarcoma tumor-bearing nude mice after i.v. injection of VACV strains at a single dose of 1×10^7^ pfu per mouse. At 7 dpvi the highest viral titers were identified in primary tumors of virus-treated mice (Fig. 6A). In the tumor tissues, there was no significant difference of the virus titers between LIVP1.1.1- and GLV-1h68-injected groups. At the same time point, we also found plaque forming units in some organs of mice injected with LIVP1.1.1 but not with GLV-1h68 (Fig. 6A). Interestingly, a few GLV-1h68 virus particles were detected in liver, lung and spleen on 35 dpvi (Fig. 6B). However, we found about 10^4^–10^5^ fold more pfus of GLV-1h68 in tumors in comparison to healthy tissues (lung, spleen, skin) at this time point (Fig. 6B).

Taken together, our data clearly demonstrated that both viruses GLV-1h68 and LIVP1.1.1 display an enhanced tumor specific replication in STSA-1 xenograft mice.

### Analysis of host immune response and the tumor vascularization in the early-phase (before 7 dpvi) of virus infection

For an investigation of the potential anti-tumor mechanism, we analyzed the virus interactions with cells of the host immune system in the early-phase (before 7 dpvi) of virus infection. Tumor single cell suspensions derived from infected and uninfected STSA-1 tumors were analyzed by flow cytometry for the presence of host immune cells at 7 dpvi (Table 2). The numbers of all tested CD11b-, MHCII-, CD19-, CD45-, F4/80-, Ly6G high, CD11c- and CD49b-positive immune cells were higher in cell suspensions derived from virus-infected tumors compared to that from uninfected controls (Table 2; ratios greater than 1). In addition, at 7 dpvi approximately 0.9% of the MHCII-positive, 1.5% of the CD45-positive and 0.26% Ly6G high^+^ cells were GFP-positive in GLV-1h68-infected tumors, indicating that either these immune cells were infected with vaccinia virus or they phagocytized virus-infected tumor cells. In this experimental setting, approximately 5- and 11-fold higher numbers of Ly6G high^+^ cells (neutrophils) were found in GLV-1h68- and LIVP1.1.1–infected tumors than in uninfected tumors, respectively (Table 2).

**Table 2:** FACS characterization and comparison of tumor single cell suspensions derived from infected and uninfected STSA-1 tumors at 7 dpvi (n = 3)

Marker	Ratio^A^ GLV-1h68/PBS	Ratio^A^ LIVP1.1.1/PBS	Positive cells
**CD11b**	1.37±0.43	1.63±1.37	granulocytes, macs, NK cells
**MHCII**	1.40±0.75	2.08±2.32	B cells, macs, DC
**CD19**	0.88±0.57	2.01±0.93	DC, B cells
**CD45**	1.16±0.58	2.16±1.60	all leukocytes
**F4/80**	1.37±0.62	2.22±3.04	majority of mature macs
**Ly6G high**	5.39±3.76	11.03±5.65	neutrophils
**CD11c**	1.83±2.82	2.03±2.09	DC, NK cells
**CD49b**	2.51±1.08	3.57±5.62	majority of NK and NKT cells
**GFP^+^**	11.15%±6.7%	n.a.	total GFP-positive cells
**GFP^+^/MHCII^+^**	0.91%±0.7%	n.a.	GFP- and MHCII-positive cells
**GFP^+^/CD45^+^**	1.52%±1.2%	n.a.	GFP- and CD45-positive cells
**GFP^+^/Ly6G**	0.26%±0.2%	n.a.	GFP- and Ly6G high-positive cells

A Ratios greater than 1 indicate an increased accumulation of host immune cells in virus-infected tumors.

Abbreviations: macs: macrophages; DC: dendritic cells; NK: natural killer cells; NKT: natural killer T cells; n.a.: not applicable.

**Figure 7:**
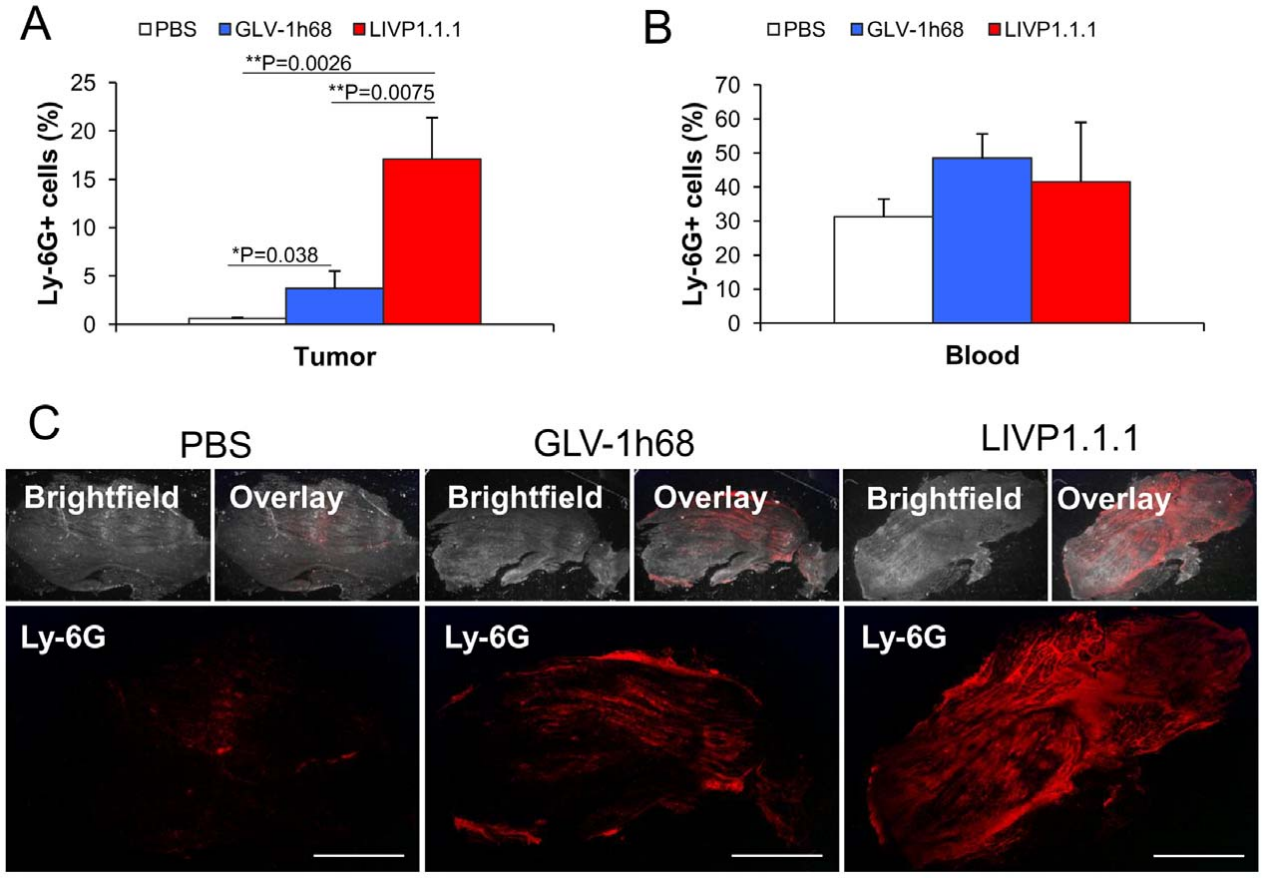
Presence of Ly-6G-positive cells (neutrophils) in virus-infected and non-infected STSA-1 xenografts at 7 dpvi. (**A, B**) Percentage of Ly-6G-positive cells (neutrophils) in tumors (**A**) and in peripheral blood (**B**) of STSA-1 xenografts 7 days after GLV-1h68-, LIVP1.1.1- or PBS-treatment. Experiments were done twice with at least 3 mice per group. The data are presented as mean values +/− standard deviations. The statistical significance was analyzed using two-way ANOVA followed Bonferroni post-test (** P<0.01, *P<0.05). (**C**) Immunohistochemical staining of Ly-6G-positive cells (neutrophils). Cryosections (10 µm-thick) of tumors were labeled with anti-Ly-6G antibody specfic for neutrophil granulocytes (red). In addition, bright-field transmission images (BF) and overlays of Ly-6G signals and transmission images (bright-field) are shown. Scale bars, 3 mm.

Since, it is known that neutrophils are one of the first cell types recruited to the sites of infections [27], we used these cells as markers for monitoring of viral infection on systemic level and in the tumor tissue. A parallel flow cytometric analysis of neutrophils was performed on peripheral blood and tumor samples. As shown in Fig. 7A there was approximately 6.6–42.1 fold higher accumulation of neutrophils in the LIVP1.1.1-treated tumors compared to GLV-1h68- and PBS-treated tumors (**P = 0.0075; **P = 0.0026), respectively. The significantly increased accumulation of neutrophils in tumors did not coincide with any reduction in peripheral blood neutrophils (Fig. 7B). An additional immunohistochemical examination confirmed the increased accumulation of neutrophils in LIVP1.1.1–infected tumors as compared to GLV-1h68-infected tumors (Fig. 7C). On the other hand, in a previous experiment, there was no significant difference of the virus titers between LIVP1.1.1- and GLV-1h68-infected tumors (Fig. 6A).

**Figure 8:**
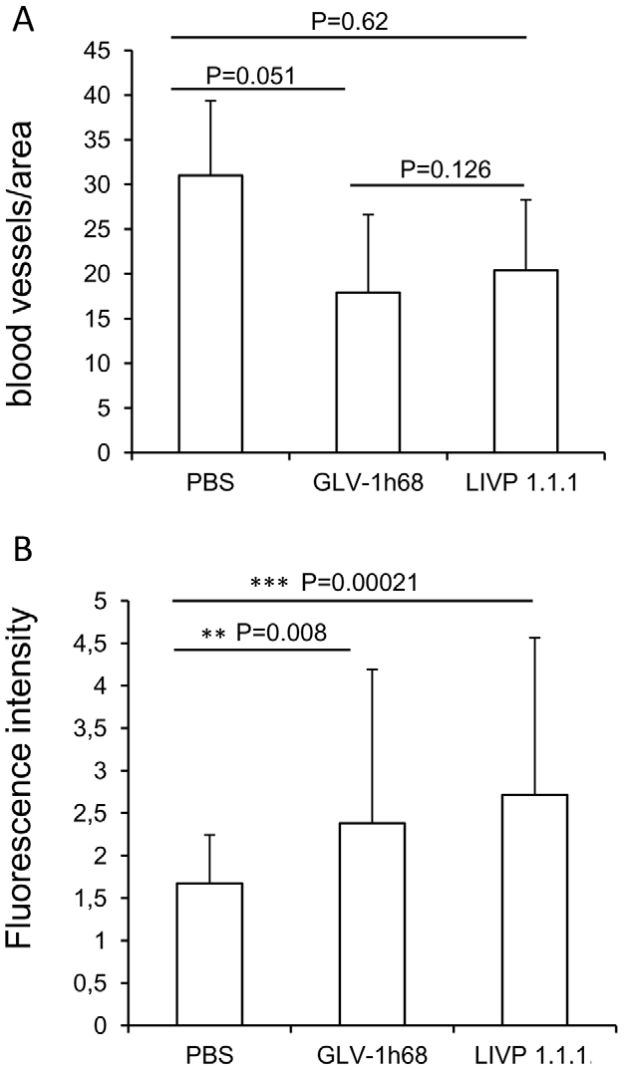
Determination of vascular density using CD31 immunohistochemistry in virus- treated and non-treated tumors at 7 dpvi. (**A**) Blood vessel density in STSA-1 tumors The vascular density was measured in CD31-labeled tumor cross-sections (n = 3 mice per group) and presented as mean values +/− standard deviations. The study was repeated in an independent experiment. There were no significant differences between groups (P>0.05, Student's t-test). (**B**) **Fluorescence intensity of the CD31 signal of blood vessels**. The fluorescence intensity of the CD31-labelling represented the average brightness of all vessel-related pixels and determined as described by [32]. The fluorescence signal was measured in 18 images of each tumor (n=3 mice per group). Shown are the mean values / standard deviations. The study was repeated in an independent experiment. ( P0.001, P0.01, Student's t-test)

To assess the impact of viral tumor colonization on the tumor vasculature, we analyzed the CD31-positive vascular network in tissue sections of the same tumors by fluorescence microscopy. In this context, CD31-labelled cross sections of control (PBS), LIPV1.1. 1- and GLV-1h68-colonized tumors were used for determination of the vascular density (Fig. 8A). The data revealed that there was no significant difference in the vascular density between the three analyzed groups (Fig. 8A). In addition, fluorescence intensity of the CD31 signal of blood vessels was measured (Fig. 8B). The results revealed that the fluorescence intensity of all vessel-related pixels of both virus-infected tumors were significantly increased in comparison to PBS-injected control tumors (PBS vs. LIVP1.1.1 *** P = 0.00021; PBS vs. GLV-1h68 **P = 0.008). This means that the virus colonization led to an up-regulation of CD31 protein, which mediates transendothelial migration of immune cells to sites of infection. In this experimental setting, however, there was no significant difference between the fluorescence intensity of vessel-related pixels of LIPV1.1.1- and GLV-1h68-tumors (P = 0.0351).

### Presence of virus and host immune cells in VACVs-infected and non-infected STSA-1 tumors at a late phase of infection

**Figure 9:**
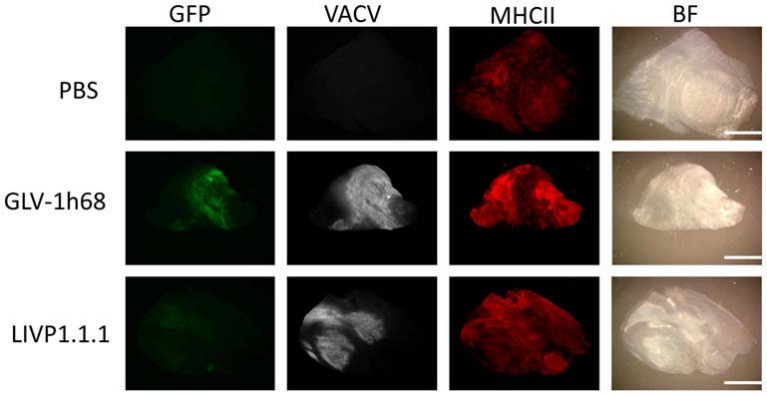
Immunohistochemical staining of infected and uninfected STSA-1 xenograft tumors at 21 dpvi. Tumor-bearing mice were either mock treated (PBS) or infected with GLV-1h68 or LIVP1.1.1. Tumor sections were labeled either with anti-vaccinia virus (VACV, grey) or anti-MHCII antibodies (red). GLV-1h68 infection and/or phagocytosis was indicated by GFP fluorescence (green). In addition, bright-field transmission images (BF) of all tumor sections are shown. Scale bars, 2.5 mm.

By immunohistolchemistry we analyzed tissue sections of primary tumors of STSA-1 tumor bearing mice injected with LIVP1.1.1, GLV-1h68 or PBS at 21 dpvi (Fig. 9). There was no significant difference in viral distribution of the two different virus strains at this stage of infection (Fig. 9, panel 2, grey color). The histological data revealed also that MHC class II-expressing host cells including B cells, macrophages and dendritic cells were present in the uninfected tumors. The single injection with LIVP1.1.1 or GLV-1h68 led to similar specific intratumoral infiltration of these host cells in the tumor tissue (Fig. 9). In summary, the results suggest that the number of the MHCII-positive immune cells in the late phase of infection is not crucial for the better oncolytic effect of LIVP1.1.1 compared to GLV-1h68 in STSA-1 xenografts.

**Figure 10:**
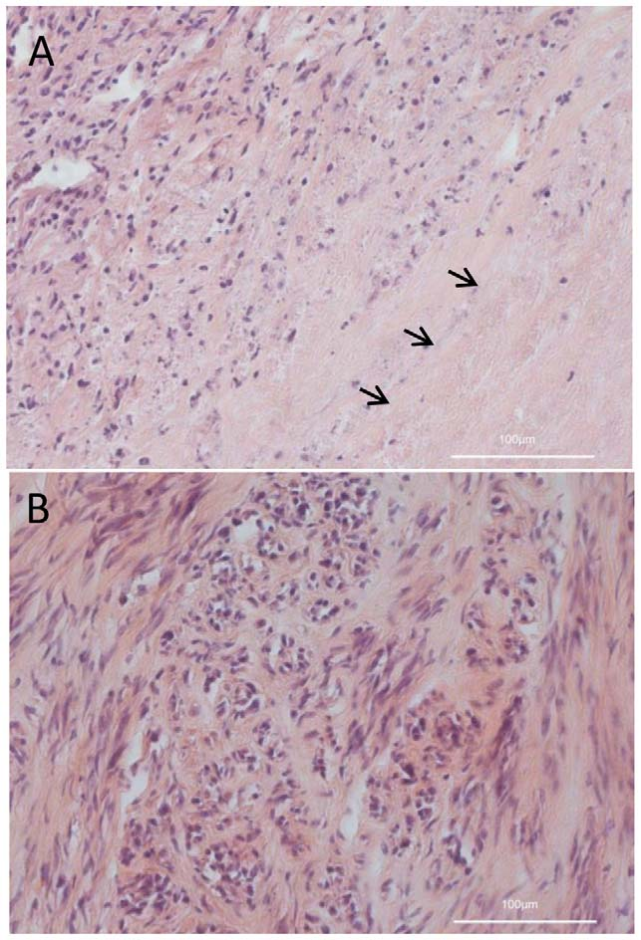
Histological analysis of LIVP1.1.1-infected (A) and non-infected (B) STSA-1 xenograft tumors, 21 dpvi (H&E, bars, 100 µm). Necrotic area is marked by arrows.

In addition, portions of the xenograft tumor tissues were paraffin-embedded, sectioned, and stained with hematoxylin and eosin (H&E) to identify possible differences between LIVP1.1.1 virus-treated (Fig. 10A) and non-treated tumors (Fig. 10B). Indeed, the virus-treated tumors looked inflamed (mixed population of neutrophils, lymphocytes and macrophages), a significant portion of the typical streaming and bundling structure of the tumor mass was disrupted and the main part of the tumor was necrotic.

## Discussion

Several oncolytic viruses including adenovirus strains CAV-1 and CAV-2 [28], canine distemper virus [29] and vaccinia virus strain GLV-1h68 [22], [23] have been used for canine cancer therapy in preclinical studies [30], [31]. However, in contrast to human studies, clinical trials with oncolytic viruses for canine cancer patients have not been reported.

In this study, we investigated the oncolytic efficiency of two vaccinia virus strains GLV-1h68 and LIVP1.1.1, against a new canine soft tissue sarcoma cell line in culture and in a xenograft model. The results showed that both VAVCs tested were able to effectively infect, replicate in and lyse canine soft tissue sarcoma cells in culture. In addition, our data revealed that the cell line STSA-1 is highly tumorigenic in nude mice. Local tumor growth occurred in 96% of the animals that had received 1×10^6^ cells subcutaneously. Interestingly, none of the control STSA-1 xenografts showed any signs of metastasis. Because the donor had evidence of lymphatic metastasis, we conclude that time for metastasis formation exceeds the time limitation owed to local tumor growth in mice. Moreover, we observed histological similarities between xenografts in mice and the pattern of the original tumor in the dog (Fig. 1). In addition, we found same indirect evidence for metastases formation in virus-treated STSA-1 mice (see later discussion). Taken together, the described xenograft model could be extremely useful as an *in vivo* tool for preclinical studies against canine soft tissue sarcoma.

The current study also demonstrated the suitability of LIVP1.1.1 and GLV-1h68, to achieve a significant inhibition of tumor growth and damage of tumor tissues in the tumor-bearing mice when compared to PBS controls. We also found that LIVP1.1.1 had a higher oncolytic potential than GLV-1h68 in these experimental settings. To clarify the reason for these differences we investigated the mechanisms by which oncolytic viruses destroy the STSA-1 tumors under these experimental conditions. Generally, it is believed that an oncolytic vaccinia virus destroys tumors by direct viral oncolysis of tumor cells [32], [33], by destruction of the tumor vasculature [34] and by induction of host antitumoral immune responses [35], or most likely, a combination of these mechanisms [18], [19].

Therefore, we first analyzed the direct viral oncolysis of STSA-1 tumor cells by examination of the virus colonization and distribution pattern of the two virus strains *in vivo*. The highest viral titers were identified in primary tumors of virus-treated mice (Fig. 6) at days 7 and 35 post virus injection. However, there was no significant difference of the tumor titers between the two viruses tested. In contrast, at 7 dpvi we also detected plaque forming units in some organs of mice injected with LIVP1.1.1 only. This fact could be evidence that the LIVP1.1.1 was either less tumor-specific or more virulent in mice compared to GLV-1h68. Surprisingly, GLV-1h68 was detected in liver, lung and spleen on 35 dpvi (Fig. 6B). In this case, the later presence of GLV-1h68 in these organs might also be a mark for metastases formation. Moreover, we have demonstrated that GLV-1h68 is a highly tumor- and metastases-selective [20], [36]. It could also be possible that due to leakiness of blood vessels in solid tumors, circulating virus-infected tumor cells or cell particles may end up in healthy tissues such as the lung, liver and spleen. However, the reason for presence of a few pfus of GLV-1h68 in some organs on 35 dpvi is currently unknown.

In summary, the differences of virus distribution after injection with GLV-1h68 or LIVP1.1.1 in STSA-1 xenografts alone could not satisfactorily explain the different oncolytic effects of these strains.

Our survival experiment revealed a significantly longer survival (P = 0.0039) of the LIVP1.1.1-treated mice compared to the PBS-treated animals. For a potential optimization of our therapeutic protocol for canine patients, an additional virus injection or combination with standard anti-cancer therapies such as chemotherapy, like demonstrated by [18], [37], are currently under investigation.

To identify other factors responsible for better oncolytic effects of LIVP1.1.1 in STSA-1 xenografts, we investigated the virus interactions with the components of the tumor microenvironment, such as tumor vasculature and the cells of the host immune system. The tumor vasculature is an important part of the tumor microenvironment supporting tumor growth by delivery of nutrients, oxygen and immune cells. Therefore, the destruction of the vascular network in tumors may be a potential therapeutic strategy for cancer therapy. In the STSA-1 xenografts model, the vascular density of tumors was not significantly changed between the GVL-1h68, LIVP1.1.1 and PBS (control) groups at 7 dpvi (Fig. 8A). In contrast, significantly increased CD31 expression in the tumor vasculature was observed at the same day after virus infection with LIPV1.1.1 and GLV-1h68 (Fig. 8B). The activated endothelium that is characterized by vascular hyperpermeability could also be one additional reason for significantly increased accumulation of host immune cells in these virus-injected tumors compared to control PBS-tumors. A similar effect on tumor vasculature was found in human breast tumor xenografts upon colonization with GLV-1h68 [32].

In the last part of our study, we investigated the virus interactions with the cells of host immune system. In this context, we analyzed the immunological host response in the early-phase (7 dpvi) of virus infection. Our flow cytometry data demonstrated a specific intratumoral infiltration of host immune cells and especially neutrophils (Ly6G high positive cells) in virus-injected STSA-1 mice after 7 dpvi (Table 2). In order to confirm these data, we have analyzed in parallel the distribution of neutrophils in tumors and peripheral blood of virus-infected and uninfected STSA-1 xenografts (Fig. 7). These experiments again revealed significantly enhanced numbers of neutrophils in tumors of virus-injected mice but not in the peripheral blood at 7 dpvi (Fig. 7). In addition, the number of neutrophils in LIVP1.1.1 infected tumors was significantly higher than that in GLV-1h68 tumors. On the basis of these data, we assume that the stronger anti-tumor effect of LIVP1.1.1 in comparison to GLV-1h68 could be dependent on the increased number of neutrophils in the tumor bed. Several other groups have reported that virotherapy induces massive tumoral infiltration of neutrophils, which may be part of virotherapy-mediated antitumor mechanism [34], [38]. In this context, Breitbach and colleagues postulated that massive neutrophil activation followed by vascular damage and apoptosis of uninfected tumor cells one day after infection are the main cause of tumor cell destruction [34]. However, it is known, that the tumor-associated neutrophils (TANs) may be associated with both pro- and anti-tumoral activities (for reviews, see [27], [39]). Recently, after a SM16 therapy, Fridlender and colleagues have identified two different populations (N1 and N2) of TANs, typical TANs (N2) that promote tumor growth and cytotoxic TANs (N1) capable of killing tumor cells [40]. In our STSA-1 model, the significantly enhanced intratumoral accumulation of neutrophils was not associated with significantly reduced vascular density in primary tumors. In these experimental settings, we found evidence for direct interactions between vaccinia virus or virus-infected cells and neutrophils in the tumor tissue (Table 2; GFP-positive cells). Therefore we speculated that high number of the virus-activated neutrophils (“N1-like”) could be cytotoxic in the tumor by providing of free oxygen radicals and proteinases in greater concentrations than typical non-activated TANs. This could be also an explanation for the better oncolytic effect of LIVP1.1.1 strain.

Taken together, however, the anti-tumor effect of virus-activated neutrophils in the virotherapy could be dependent on type and origin of tumors, stage of tumor development, virus strain or host studied, but finally on the balance between antiviral and antitumoral immune responses.

Our findings suggest that the virotherapy-mediated anti-tumor mechanism in STSA-1 xenografts could be a combination of the direct viral oncolysis of tumor cells and the virus-dependent activation of tumor-associated host immune cells, mainly neutrophils.

In summary, therapy with the vaccinia strains and especial LIVP1.1.1 demonstrated outstanding anti-tumor activity in canine soft tissue sarcoma cells and in the STSA-1 xenograft model. Therefore we propose that the LIVP1.1.1 vaccinia virus strain may be useful for the treatment of spontaneous soft-tissue sarcomas in dogs.

## Supporting Information

Figure S1 **Cytocentrifuged STSA-1 cells magnification ×500.** (**A**) **Wright-Giemsa stain,** (**B**) **vimentin,** (**C**) **ALP activity,** (**D**) **CD18,** (**E**) **cytokeratin.** Brown coloration is indicative of positive staining. Negatively staining samples were counterstained with Wright-Giemsa stain to visualize cells. All positive and negative controls stained adequately (data not shown).
(TIF)

Figure S2 **Short tandem repeat (STR) analysis of STSA-1 cells.** The validation of the cell line was preformed as recently described [25].The STK kit (StockMarks for Dogs: Canine Genotyping Kit, Applied Biosystems, Foster City, CA) tested 10 different loci (**A**): PEZ 1, FHC 2054, FHC 2010; (**B**) PEZ 5, PEZ 20; PEZ 12; (**C**) PEZ 3, PEZ 6, PEZ 8, FHC 2079.The observed allele sizes were typical for canine cells.
(TIF)
