# Supplementary figures and images for: Preclinical Evaluation of Oncolytic Vaccinia Virus for Therapy of Canine Soft Tissue Sarcoma
Source: PLoS One. 2012 May 15;7(5):e37239. doi: 10.1371/journal.pone.0037239 (PMC3352892; doi:10.1371/journal.pone.0037239)

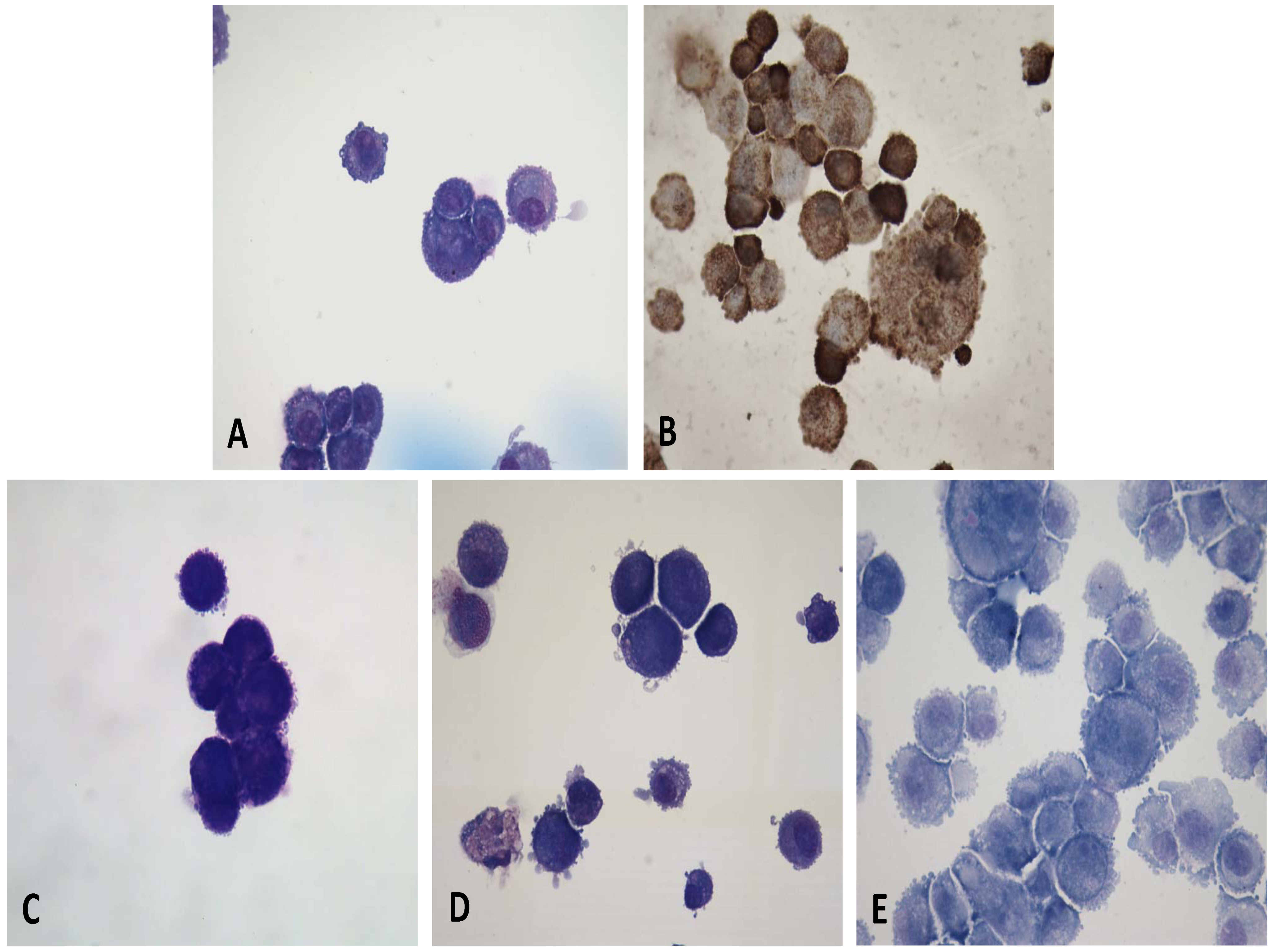

Supplement: Figure S1 — Cytocentrifuged STSA-1 cells magnification ×500. (A) Wright-Giemsa stain, (B) vimentin, (C) ALP activity, (D) CD18, (E) cytokeratin. Brown coloration is indicative of positive staining. Negatively staining samples were counterstained with Wright-Giemsa stain to visualize cells. All positive and negative controls stained adequately (data not shown). (TIF) [file pone.0037239.s001.tif]

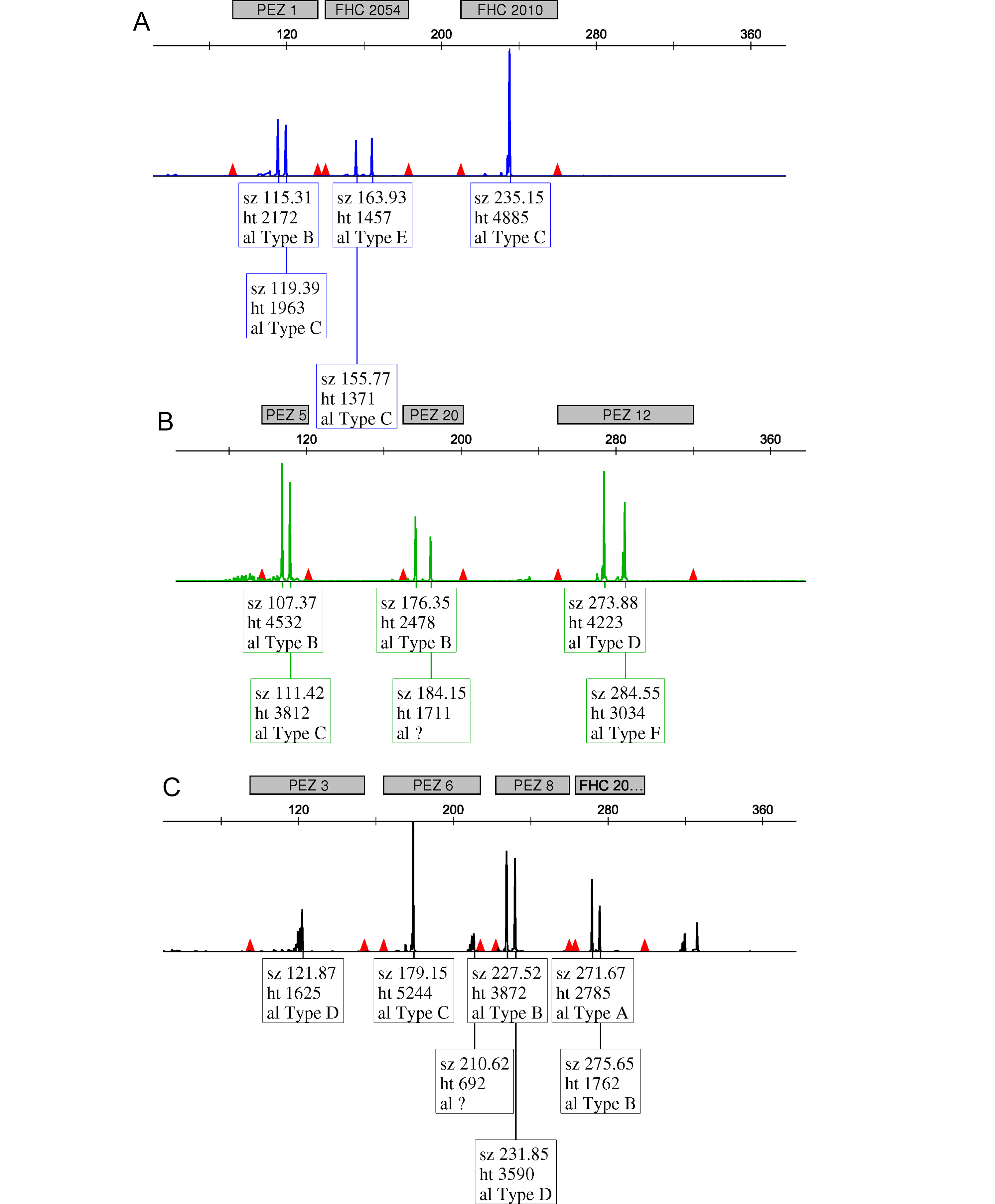

Supplement: Figure S2 — Short tandem repeat (STR) analysis of STSA-1 cells. The validation of the cell line was preformed as recently described [25].The STK kit (StockMarks for Dogs: Canine Genotyping Kit, Applied Biosystems, Foster City, CA) tested 10 different loci (A): PEZ 1, FHC 2054, FHC 2010; (B) PEZ 5, PEZ 20; PEZ 12; (C) PEZ 3, PEZ 6, PEZ 8, FHC 2079.The observed allele sizes were typical for canine cells. (TIF) [file pone.0037239.s002.tif]
